# Supplementary material for: Systemic Immune-Inflammation Index Predicted Short-Term Outcomes in Patients Undergoing Isolated Tricuspid Valve Surgery
Source: J Clin Med. 2021 Sep 14;10(18):4147. doi: 10.3390/jcm10184147 (PMC8471776; doi:10.3390/jcm10184147)
Supplement: Supplementary file 1 [file jcm-10-04147-s001.zip › Supplementary table_R.pdf]

Supplementary Table S1. Predictors associated with composite 30-day major postoperative complications in patients undergoing isolated tricuspid valve surgery.

Variables	Univariate		Multivariable	
	Hazard Ratio (95% CI)	*P* value	Hazard Ratio (95% CI)	*P* value
**SII (≥1423.12×10⁹/L)**	4.96 (2.73–9.01)	**0.000**	2.63 (1.18–5.84)	**0.018**
Sex (female)	1.06 (0.67–1.66)	0.809		
Age, yr	1.03 (1.01–1.05)	**0.003**		
Body mass index, kg/m²	0.96 (0.90–1.04)	0.328		
Diabetes	1.71 (1.01–2.88)	**0.046**		
Hypertension	1.63 (1.05–2.54)	**0.029**		
NYHA	1.73 (1.12–2.67)	**0.014**		
EuroSCORE	1.06 (1.03–1.10)	**0.001**		
Preoperative hematocrit, %	0.93 (0.88–0.96)	**0.001**		
Total bilirubin, mg/dl	1.06 (0.70–1.60)	0.790		
Serum albumin, g/dL	0.43 (0.29–0.62)	**0.001**		
Creatinine, mg/dL	0.96 (0.76–1.21)	0.718		
AST, IU/L	1.00 (0.98–1.03)	0.920		
ALT, IU/L	0.98 (0.96–1.01)	0.152		
ALP, IU/L	1.01 (1.00–1.01)	**0.020**		
Uric acid, mg/dL	1.09 (0.98–1.20)	0.095		
BNP, pg/mL	1.00 (1.00–1.01)	0.780		
CRP	1.39 (1.18–1.64)	**0.001**		
PLR	1.04 (1.03–1.06)	**0.001**		
NLR	1.33 (1.24–1.42)	**0.001**		
LVEF, %	0.98 (0.98–1.01)	0.106		

RV dysfunction	1.74 (1.10–2.73)	**0.017**		
PASP, mmHg	0.99 (0.98–1.01)	0.293		
Use of ACEI/ARB	1.82 (1.17–2.81)	**0.007**		
Use of β-blocker	1.74 (1.11–2.71)	**0.015**		
Use of CCB	2.03 (1.30–3.17)	**0.002**		
Use of Digoxin	1.34 (0.85–2.12)	0.210		
Use of Diuretics	1.34 (1.85–2.16)	0.210		

Supplementary Table S2. Predictors associated with composite 30-day major postoperative complications in patients undergoing isolated tricuspid valve surgery.

Variables	Univariate		Multivariable	
	Hazard Ratio (95% CI)	*P* value	Hazard Ratio (95% CI)	*P* value
**SII (≥878.06×10⁹/L)**	4.91 (2.95–8.16)	**0.000**	2.19 (1.05–4.54)	**0.036**
Sex (female)	1.06 (0.67–1.66)	0.809		
Age, yr	1.03 (1.01–1.05)	**0.003**		
Body mass index, kg/m²	0.96 (0.90–1.04)	0.328		
Diabetes	1.71 (1.01–2.88)	**0.046**		
Hypertension	1.63 (1.05–2.54)	**0.029**		
NYHA	1.73 (1.12–2.67)	**0.014**		
EuroSCORE	1.06 (1.03–1.10)	**0.001**		
Preoperative hematocrit, %	0.93 (0.88–0.96)	**0.001**		
Total bilirubin, mg/dl	1.06 (0.70–1.60)	0.790		
Serum albumin, g/dL	0.43 (0.29–0.62)	**0.001**		
Creatinine, mg/dL	0.96 (0.76–1.21)	0.718		
AST, IU/L	1.00 (0.98–1.03)	0.920		

ALT, IU/L	0.98 (0.96–1.01)	0.152
ALP, IU/L	1.01 (1.00–1.01)	**0.020**
Uric acid, mg/dL	1.09 (0.98–1.20)	0.095
BNP, pg/mL	1.00 (1.00–1.01)	0.780
CRP	1.39 (1.18–1.64)	**0.001**
PLR	1.04 (1.03–1.06)	**0.001**
NLR	1.33 (1.24–1.42)	**0.001**
LVEF, %	0.98 (0.98–1.01)	0.106
RV dysfunction	1.74 (1.10–2.73)	**0.017**
PASP, mmHg	0.99 (0.98–1.01)	0.293
Use of ACEI/ARB	1.82 (1.17–2.81)	**0.007**
Use of β-blocker	1.74 (1.11–2.71)	**0.015**
Use of CCB	2.03 (1.30–3.17)	**0.002**
Use of Digoxin	1.34 (0.85–2.12)	0.210
Use of Diuretics	1.34 (1.85–2.16)	0.210

Supplementary Table S3. Predictors associated with composite 30-day major postoperative complications in patients undergoing isolated tricuspid valve surgery.

Variables	Univariate		Multivariable	
	Hazard Ratio (95% CI)	*P* value	Hazard Ratio (95% CI)	*P* value
**SII (≥750×10^9/L)**	5.34 (3.37–8.47)	**0.000**	2.81 (1.37–5.76)	**0.005**
Sex (female)	1.06 (0.67–1.66)	0.809		
Age, yr	1.03 (1.01–1.05)	**0.003**		
Body mass index, kg/m^2	0.97 (0.90–1.04)	0.328		
Diabetes	1.71 (1.01–2.88)	**0.046**		

Hypertension	1.63 (1.05–2.54)	**0.029**
NYHA	1.73 (1.12–2.67)	**0.014**
EuroSCORE	1.06 (1.03–1.10)	**0.001**
Preoperative hematocrit, %	0.93 (0.90–0.96)	**0.001**
Total bilirubin, mg/dl	1.17 (0.83–1.66)	0.365
Serum albumin, g/dL	0.52 (0.37–0.74)	**0.001**
Creatinine, mg/dL	0.96 (0.80–1.17)	0.737
AST, IU/L	1.00 (0.98–1.03)	0.920
ALT, IU/L	0.98 (0.96–1.01)	0.152
ALP, IU/L	1.05 (1.00–1.01)	**0.020**
Uric acid, mg/dL	1.09 (0.99–1.20)	0.095
BNP, pg/mL	1.00 (1.00–1.01)	0.200
CRP	1.39 (1.18–1.64)	**0.001**
PLR	1.04 (1.03–1.06)	**0.001**
NLR	1.33 (1.24–1.42)	**0.001**
LVEF, %	0.98 (0.95–1.01)	0.106
RV dysfunction	1.74 (1.10–2.73)	**0.017**
PASP, mmHg	0.99 (0.98–1.01)	0.293
Use of ACEI/ARB	1.82 (1.17–2.81)	**0.007**
Use of β-blocker	1.74 (1.11–2.71)	**0.015**
Use of CCB	2.03 (1.30–3.17)	**0.002**
Use of Digoxin	1.34 (0.85–2.12)	0.210
Use of Diuretics	1.34 (1.85–2.16)	0.210

Supplementary Table S4. Predictors associated with composite 30-day major postoperative complications in patients undergoing isolated tricuspid valve surgery.

Variables	Univariate		Multivariable	
	Hazard Ratio (95% CI)	*P* value	Hazard Ratio (95% CI)	*P* value
**SII (≥694.03×10⁹/L)**	5.63 (3.60–8.80)	**0.000**	3.97 (1.72–9.19)	**0.001**
Sex (female)	1.06 (0.67–1.66)	0.809		
Age, yr	1.03 (1.01–1.05)	**0.003**		
Body mass index, kg/m^2	0.97 (0.90–1.04)	0.328		
Diabetes	1.71 (1.01–2.88)	**0.046**		
Hypertension	1.63 (1.05–2.54)	**0.029**		
NYHA	1.73 (1.12–2.67)	**0.014**		
EuroSCORE	1.06 (1.03–1.10)	**0.001**		
Preoperative hematocrit, %	0.93 (0.90–0.96)	**0.001**		
Total bilirubin, mg/dl	1.17 (0.83–1.66)	0.365		
Serum albumin, g/dL	0.52 (0.37–0.74)	**0.001**		
Creatinine, mg/dL	0.96 (0.80–1.17)	0.737		
AST, IU/L	1.00 (0.98–1.03)	0.920		
ALT, IU/L	0.98 (0.96–1.01)	0.152		
ALP, IU/L	1.05 (1.00–1.01)	**0.020**		
Uric acid, mg/dL	1.09 (0.99–1.20)	0.095		
BNP, pg/mL	1.00 (1.00–1.01)	0.200		
CRP	1.39 (1.18–1.64)	**0.001**		
PLR	1.04 (1.03–1.06)	**0.001**		
NLR	1.33 (1.24–1.42)	**0.001**		
LVEF, %	0.98 (0.95–1.01)	0.106		

RV dysfunction	1.74 (1.10–2.73)	**0.017**		
PASP, mmHg	0.99 (0.98–1.01)	0.293		
Use of ACEI/ARB	1.82 (1.17–2.81)	**0.007**		
Use of β-blocker	1.74 (1.11–2.71)	**0.015**		
Use of CCB	2.03 (1.30–3.17)	**0.002**		
Use of Digoxin	1.34 (0.85–2.12)	0.210		
Use of Diuretics	1.34 (1.85–2.16)	0.210		

Supplementary Table S5. Predictors associated with composite 30-day major postoperative complications in patients undergoing isolated tricuspid valve surgery.

Variables	Univariate		Multivariable	
	Hazard Ratio (95% CI)	*P* value	Hazard Ratio (95% CI)	*P* value
**SII (≥551×10^9/L)**	4.28 (2.76–6.62)	**0.000**	2.12 (1.11–4.08)	**0.024**
Sex (female)	1.06 (0.67–1.66)	0.809		
Age, yr	1.03 (1.01–1.05)	**0.003**		
Body mass index, kg/m^2	0.96 (0.90–1.04)	0.328		
Diabetes	1.71 (1.01–2.88)	**0.046**		
Hypertension	1.63 (1.05–2.54)	**0.029**		
NYHA	1.73 (1.12–2.67)	**0.014**		
EuroSCORE	1.06 (1.03–1.10)	**0.001**		
Preoperative hematocrit, %	0.93 (0.88–0.96)	**0.001**		
Total bilirubin, mg/dl	1.06 (0.70–1.60)	0.790		
Serum albumin, g/dL	0.43 (0.29–0.62)	**0.001**		
Creatinine, mg/dL	0.96 (0.76–1.21)	0.718		
AST, IU/L	1.00 (0.98–1.03)	0.920		

ALT, IU/L	0.98 (0.96–1.01)	0.152
ALP, IU/L	1.01 (1.00–1.01)	**0.020**
Uric acid, mg/dL	1.09 (0.98–1.20)	0.095
BNP, pg/mL	1.00 (1.00–1.01)	0.780
CRP	1.39 (1.18–1.64)	**0.001**
PLR	1.04 (1.03–1.06)	**0.001**
NLR	1.33 (1.24–1.42)	**0.001**
LVEF, %	0.98 (0.98–1.01)	0.106
RV dysfunction	1.74 (1.10–2.73)	**0.017**
PASP, mmHg	0.99 (0.98–1.01)	0.293
Use of ACEI/ARB	1.82 (1.17–2.81)	**0.007**
Use of β-blocker	1.74 (1.11–2.71)	**0.015**
Use of CCB	2.03 (1.30–3.17)	**0.002**
Use of Digoxin	1.34 (0.85–2.12)	0.210
Use of Diuretics	1.34 (1.85–2.16)	0.210

Supplementary Table S6. Predictors associated with composite 30-day major postoperative complications in patients undergoing isolated tricuspid valve surgery.

Variables	Univariate		Multivariable	
	Hazard Ratio (95% CI)	*P* value	Hazard Ratio (95% CI)	*P* value
**SII (≥326.26×10⁹/L, median)**	2.54 (1.58–4.09)	**0.000**	2.13 (1.17–3.89)	**0.014**
Sex (female)	1.06 (0.67–1.66)	0.809		
Age, yr	1.03 (1.01–1.05)	**0.003**		
Body mass index, kg/m²	0.96 (0.90–1.04)	0.328		
Diabetes	1.71 (1.01–2.88)	**0.046**		

Hypertension	1.63 (1.05–2.54)	**0.029**
NYHA	1.73 (1.12–2.67)	**0.014**
EuroSCORE	1.06 (1.03–1.10)	**0.001**
Preoperative hematocrit, %	0.93 (0.88–0.96)	**0.001**
Total bilirubin, mg/dl	1.06 (0.70–1.60)	0.790
Serum albumin, g/dL	0.43 (0.29–0.62)	**0.001**
Creatinine, mg/dL	0.96 (0.76–1.21)	0.718
AST, IU/L	1.00 (0.98–1.03)	0.920
ALT, IU/L	0.98 (0.96–1.01)	0.152
ALP, IU/L	1.01 (1.00–1.01)	**0.020**
Uric acid, mg/dL	1.09 (0.98–1.20)	0.095
BNP, pg/mL	1.00 (1.00–1.01)	0.780
CRP	1.39 (1.18–1.64)	**0.001**
PLR	1.04 (1.03–1.06)	**0.001**
NLR	1.33 (1.24–1.42)	**0.001**
LVEF, %	0.98 (0.98–1.01)	0.106
RV dysfunction	1.74 (1.10–2.73)	**0.017**
PASP, mmHg	0.99 (0.98–1.01)	0.293
Use of ACEI/ARB	1.82 (1.17–2.81)	**0.007**
Use of β-blocker	1.74 (1.11–2.71)	**0.015**
Use of CCB	2.03 (1.30–3.17)	**0.002**
Use of Digoxin	1.34 (0.85–2.12)	0.210
Use of Diuretics	1.34 (1.85–2.16)	0.210
