# Supplementary figures and images for: Systemic Immune-Inflammation Index Predicted Short-Term Outcomes in Patients Undergoing Isolated Tricuspid Valve Surgery
Source: J Clin Med. 2021 Sep 14;10(18):4147. doi: 10.3390/jcm10184147 (PMC8471776; doi:10.3390/jcm10184147)

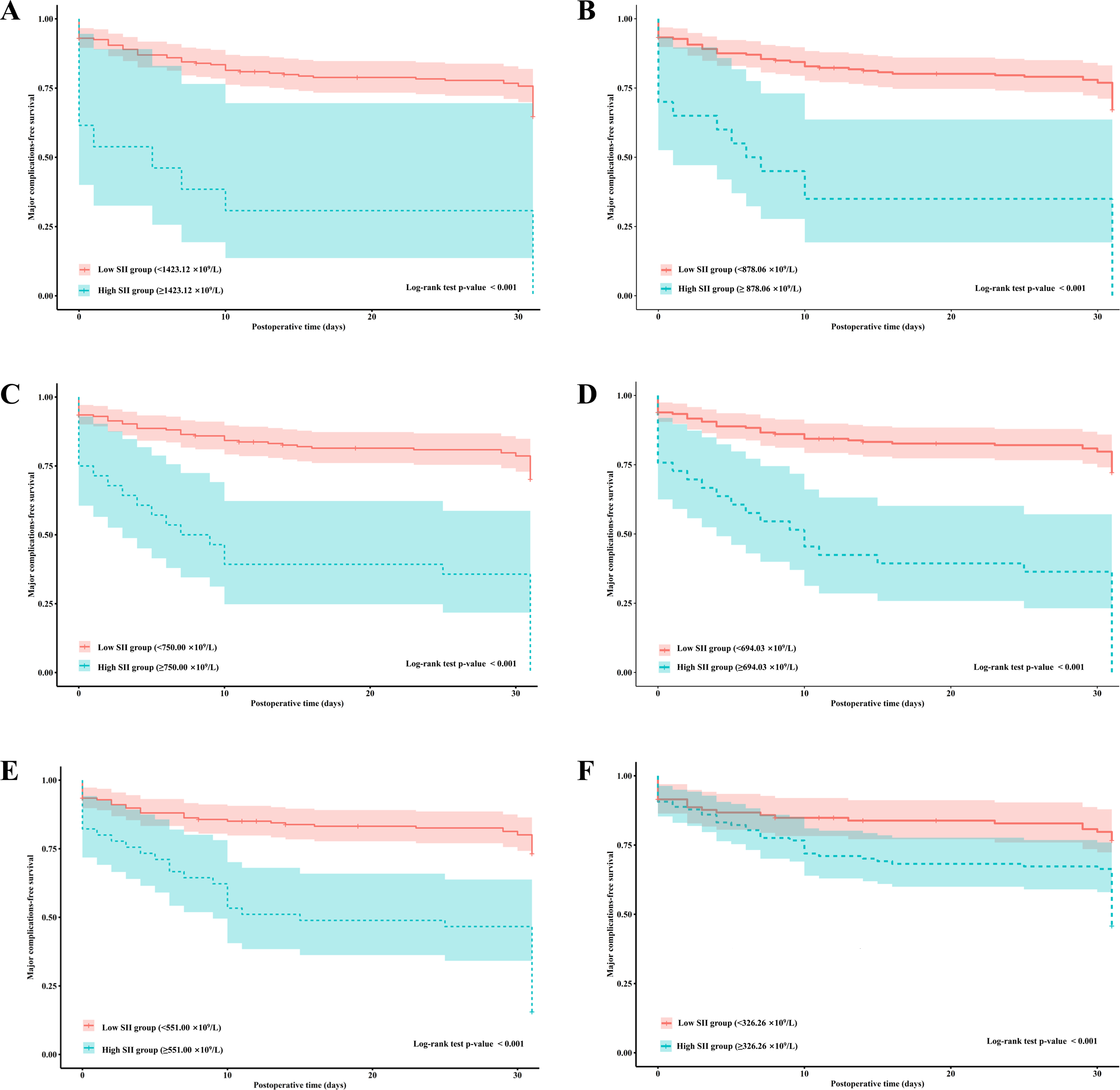

Supplement: Supplementary file 1 [file jcm-10-04147-s001.zip › Supplementary Figure_R.tif]
